# Diagnostic yield of nitroglycerin-potentiated head-up tilt test in a pediatric population with suspected reflex syncope

**DOI:** 10.1007/s10286-025-01145-5

**Published:** 2025-08-04

**Authors:** Vincenzo Russo, Angelo Comune, Giangiacomo Di Nardo, Erika Parente, Giovanni Maria Di Marco, Angelica De Nigris, Maria Giovanna Russo, Berardo Sarubbi, Gerardo Nigro, Michele Brignole

**Affiliations:** 1 Cardiology and Syncope Unit, Department of Translational Medical Sciences, University of Campania Luigi Vanvitelli, Monaldi Hospital, Via Leonardo Bianchi, Naples, Italy; 2 Department of Pediatric Cardiology, Santobono-Pausilipon Children’s Hospital, Naples, Italy; 3 Pediatric Cardiology Unit, University of Campania Luigi Vanvitelli, Azienda Ospedaliera di Rilievo Nazionale (AORN) Ospedali dei Colli, Monaldi Hospital, Naples, Italy; 4 Adult Congenital Heart Disease Unit, AORN Ospedali dei Colli, Monaldi Hospital, Naples, Italy; 5 Cardiology Unit and Department of Cardiology, S. Luca Hospital, Istituti di Ricovero e Cura a Carattere Scientifico (IRCCS) Istituto Auxologico Italiano, Faint and Fall Research Centre, Milan, Italy

**Keywords:** Syncope, Reflex syncope, Situational, Hypotension, Bradycardia, Tilt testing, Children, Asystole, Vasodepression

## Abstract

**Background:**

Syncope is a prevalent issue in pediatric patients. The nitroglycerin (NTG)-potentiated head-up tilt test (HUTT) is widely used in adults for diagnosing reflex syncope; however, few and contrasting data are available in pediatric populations. The aim of our study was to evaluate the positivity rate and types of responses to NTG-potentiated HUTT in pediatric patients with suspected reflex syncope.

**Methods:**

We conducted a retrospective multicenter analysis of 307 pediatric patients (mean age: 14.4 ± 2.8 years; 57.6% female) who underwent HUTT at two syncope units in Naples, Italy. A group of 16 healthy pediatric subjects (13 ± 3.2 years; 37.5% female) with no history of syncope was used as a control. We described the HUTT overall positivity rate and responses; moreover, the positivity rate, sensitivity, and specificity were evaluated. A multivariate analysis was performed to test the association of positive response to HUTT with a set of clinical covariates.

**Results:**

The overall HUTT positivity rate was 74.9%, ranging from 51.5% to 81.6% among pediatric patients with non-classical and classical presentation, respectively. The HUTT positivity rate among healthy control group was 18.7%; consequently the HUTT specificity was 81.3%. Younger age (OR: 0.84; *p* = 0.005) and female sex (OR: 2.3; *p* = 0.005) were independent predictors of HUTT positivity; in contrast, the non-classical presentation of syncope (OR: 0.23; *p* < 0.001) and situational syncope (OR: 0.2; *p* = 0.006) correlated negatively with HUTT positivity.

**Conclusions:**

NTG-potentiated HUTT showed a high positivity rate, good sensitivity, and specificity in pediatric patients with suspected reflex syncope. Some patients and syncope-related features independently correlated with HUTT positivity. Cardioinhibitory response was more prevalent in pediatric patients with a non-classical presentation of reflex syncope.

## Introduction

Syncope is a common clinical issue in the pediatric age group. Up to 20% of children have experienced at least one episode of transient loss of consciousness (TLOC) by the end of the adolescence [1]; moreover, about 1% of total admissions to the pediatric emergency department are due to syncope [2]. The head-up tilt test (HUTT) is a valuable diagnostic tool for assessing patients with suspected reflex syncope. Among various protocols, nitroglycerin (NTG)-potentiated ones showed the highest sensitivity—up to 66%—in adult patients [3, 4]. There are limited and conflicting data about the diagnostic yield of HUTT in pediatric populations [5–7] owing to differences in the selection of study populations, HUTT protocols, and outcomes. Our aim was to evaluate the positivity rate and types of responses to NTG-potentiated HUTT in pediatric patients with suspected reflex syncope and, furthermore, to compare these findings with those of a control pediatric population without a history of syncope.

## Materials and methods

We retrospectively assessed the positivity rate of NTG-potentiated HUTT in consecutive patients aged < 18 years who underwent the test for suspected reflex syncope at two pediatric syncope units (University of Campania Luigi Vanvitelli, Monaldi Hospital, in Naples and Santobono-Pausillipon Hospital in Naples), from 1 March 2017 to 31 December 2023. All patients underwent a comprehensive clinical evaluation, including careful history taking concerning present and previous attacks, physical examination, supine and standing manual blood pressure (BP) measurements, and 12-lead electrocardiogram (ECG). Moreover, on the basis of the results of the initial evaluation, ECG monitoring and echocardiogram were performed when indicated [8] to exclude cardiac syncope. After a comprehensive clinical evaluation, patients were classified as classical or non-classical vasovagal syncope (VVS). The classical VVS was characterized by precipitating triggers such as emotional distress (emotions, fear, severe pain, disgust, medical setting) or orthostatic stress (prolonged standing) and prodromal symptoms owing to activation of the autonomic system (nausea, vomiting, abdominal discomfort, pallor, and sweating) or owing to cerebral and retinal hypoperfusion (dizziness, light-headedness, and blurred vision). The suspected non-classical VVS included episodes with uncertain or apparently absent triggers and/or premonitory symptoms when other causes of syncope were excluded at initial comprehensive clinical evaluation before HUTT. Patients with other diagnoses than suspected reflex syncope (i.e., pseudopsycogen syncope or postural orthostatic tachycardia syndrome) were excluded from the analysis. The HUTT was performed according to the “Italian Protocol,” with a supine pretilt phase of 10 min without venous cannulation, followed by a 20-min passive phase at 70 °C. In the case of negativity, a NTG challenge with a fixed dose of 300 µg was sublingually administered with the patient in the upright position [9]. From 2021, the “Italian Fast Protocol” was systematically used, featuring shorter passive and active phases (10 min, respectively) [10]. The HUTT continued until complete loss of consciousness (LOC) occurred, indicated by a lack of response to vocal stimuli, loss of muscle tonus, and jerking movements, whichever occurred first, or completion of the protocol; the maximum time required for tilting down the motorized tilt table was 12 s. During the whole duration of the HUTT, continuous electrocardiographic monitoring and noninvasive beat-to-beat arterial blood pressure measurement (Task Force@ monitor; CNSystem, Graz, Austria) was performed. The positive responses were classified according to the new Vasovagal Syncope International Study (VASIS) classification [11], which was also suggested by the 2018 European Society of Cardiology Guidelines [8]. In particular, vasodepressive (VD) response was defined when syncope occurred during hypotension, along with slight or no decrease (< 10% bpm) decrease in heart rate at the time of HUTT-induced syncope compared with the peak value of heart rate reached during the presyncopal phase of the test. A response was classified as cardioinhibitory when syncope occurred in the presence of a ventricular pause of > 3 s; meanwhile mixed response was established when syncope occurred along with bradycardia (decrease in heart rate > 10%) and hypotension. As a secondary end point, the study population was divided into two groups on the basis of positive or negative response to HUTT. These two groups were compared to identify any underlying clinical characteristics that might predict the HUTT positivity rate. The HUTT specificity was calculated in a group of healthy pediatric patients with no history of transient loss of consciousness (TLOC; control group) who were screened for sports participation and who voluntarily accepted undergoing the HUTT, after written informed consent was obtained from their parents and/or legal guardians in accordance with ethical standards. This study was conducted according to the Declaration of Helsinki and approved by the institutional ethics committees (ID-168/02032021) of University of Campania Luigi Vanvitelli; written informed consent for data collection was obtained from the patient’s parents or legal guardians.

### Statistical analysis

The normal distribution of the data was assessed using the Kolmogorov–Smirnov and Shapiro–Wilk tests. Normally distributed variables were expressed as mean ± standard deviation (SD), whereas non-normal distributed ones were expressed as the median and interquartile range (IQR). Categorical variables were reported as numbers and percentages. Continuous normally distributed variables were compared by using the Student *t*-test; differences between abnormally distributed variables were tested with the Mann–Whitney *U* test. Categorical variables were compared with a chi-squared test, or Fisher exact test, when appropriate. Those baseline clinical characteristics that were associated with HUTT positivity at univariate analysis with a *p*-value < 0.05 were inserted and analyzed in a stepwise multivariable logistic regression model. Results were presented as an odds ratio (OR) with a 95% confidence interval (CI) for each covariate in the model. An *α* level of 0.05 with Bonferroni correction for the number of hypotheses was considered significant for all measurements. All statistical analyses were performed using STATA software (version 11.1, College Station, TX: StataCorp LLC).

## Results

### Study population

The study population consisted of 307 patients (14.4 ± 2.8 years; age range 7–17 years; 57.6% female), of whom 230 (74.9%) showed a positive HUTT response with reproduction of their spontaneous symptomatology. The clinical characteristics of the study population are presented in Table 1. Among patients with classical presentation (*n* = 239; 77.9%), the HUTT positivity rate was 81.6%; in contrast, in those with non-classical presentation (*n* = 68; 22.1%), it reached 51.5%. A total of 69 patients (22.5%) experienced syncope during orthostatic passive phase and the remaining (*n* = 161; 52.4%) after NTG administration. The mixed response was the most prevalent (67.8%), followed by the cardioinhibitory (23.9%) and the vasodepressive response (8.3%). The relative prevalence of cardioinhibitory response was higher among patients with non-classical presentation compared with those with classical presentation (54.3% versus 18.5%; *p* < 0.0001). The control group consisted of 16 healthy subjects (13 ± 3.2 years; 37.5% female), with 3 patients (18.7%) having a positive HUTT response (false positive); the remaining subjects (*n* = 13; 81.3%) showed negative HUTT responses (true negative). There were no differences in baseline clinical characteristics between the syncope and healthy populations (Table 2).

**Table 1:** Baseline characteristics of the study population dichotomized by response to HUTT

	Overall population *n* = 307	Positive responses *n* = 230	Negative responses *n* = 77	*p*-Value
Age (years), mean ± SD	14.4 ± 2.8	14 ± 2.8	15.7 ± 2.4	< 0.0001
Female, *n* (%)	177 (57.6%)	142 (61.7%)	35 (45.5%)	0.001
BMI, mean ± SD	22.2 ± 3.4	22.1 ± 2.9	22.6 ± 4.7	0.25
SBP (recumbent) (mmHg), mean ± SD	116.4 ± 10.6	115.4 ± 9.5	119.05 ± 13	0.01
DBP (recumbent) (mmHg), mean ± SD	66.1 ± 9	65.5 ± 7.8	67.6 ± 11.8	0.09
HR (recumbent) (bpm), mean ± SD	81 ± 16.2	82.2 ± 15.1	77.5 ± 18.9	0.03
Smoker, *n* (%)	10 (3.2%)	9 (3.9%)	1 (1.3%)	0.26
Hypertension, *n* (%)	1 (0.3%)	1 (0.4%)	0	0.6
ACE-i, *n* (%)	1 (0.3%)	1 (0.4%)	0	0.6
Diuretics, *n* (%)	1 (0.3%)	0	1 (1.3%)	0.08
Number of syncopes (*n*), mean ± SD	2.98 ± 2.65	3 ± 2.7	3 ± 2.9	0.99
Traumatic syncope, *n* (%)	39 (12.7%)	31 (13.5%)	8 (10.4%)	0.5
Classical, *n* (%)	239 (77.9%)	195 (84.8%)	44 (57.1%)	< 0.0001
Nonclassical, *n* (%)	68 (22.1%)	35 (15.2%)	33 (42.9%)	< 0.0001
Situational syncope, *n* (%)	15 (4.9%)	7 (3%)	8 (10.4%)	0.009

*BMI* body mass index, *SBP* systolic blood pressure, *DBP* diastolic blood pressure, *HR* heart rate, *ACE-i* angiotensin-converting enzyme inhibitors

**Table 2:** Baseline characteristics of healthy subjects compared with the population with syncope

	Population with syncope *n* = 307	Healthy subjects *n* = 16	*p*-Value
Age (years), mean ± SD	14.4 ± 2.8	13 ± 3.2	0.053
Female, *n* (%)	177 (57.6%)	6 (37.5%)	0.11
BMI, mean ± SD	22.2 ± 3.4	21.1 ± 1.2	0.19
Smoker, *n* (%)	10 (3.2%)	1 (6.2%)	0.5
Hypertension, *n* (%)	1 (0.3%)	0	0.8
ACE-i, *n* (%)	1 (0.3%)	0	0.8
Diuretics, *n* (%)	1 (0.3%)	0	0.8
SBP (recumbent), mmHg	116.4 ± 10.6	117.7 ± 14.4	0.64
DBP (recumbent) (mmHg)	66.1 ± 9	68.8 ± 12.4	0.25
HR (recumbent) (bpm)	81 ± 16.2	74.5 ± 14.4	0.11

*BMI* body mass index, *SBP* systolic blood pressure, *DBP* diastolic blood pressure, *HR* heart rate, *ACE-i* angiotensin converting enzyme inhibitors

### Predictors of HUTT positivity

**Fig. 1:**
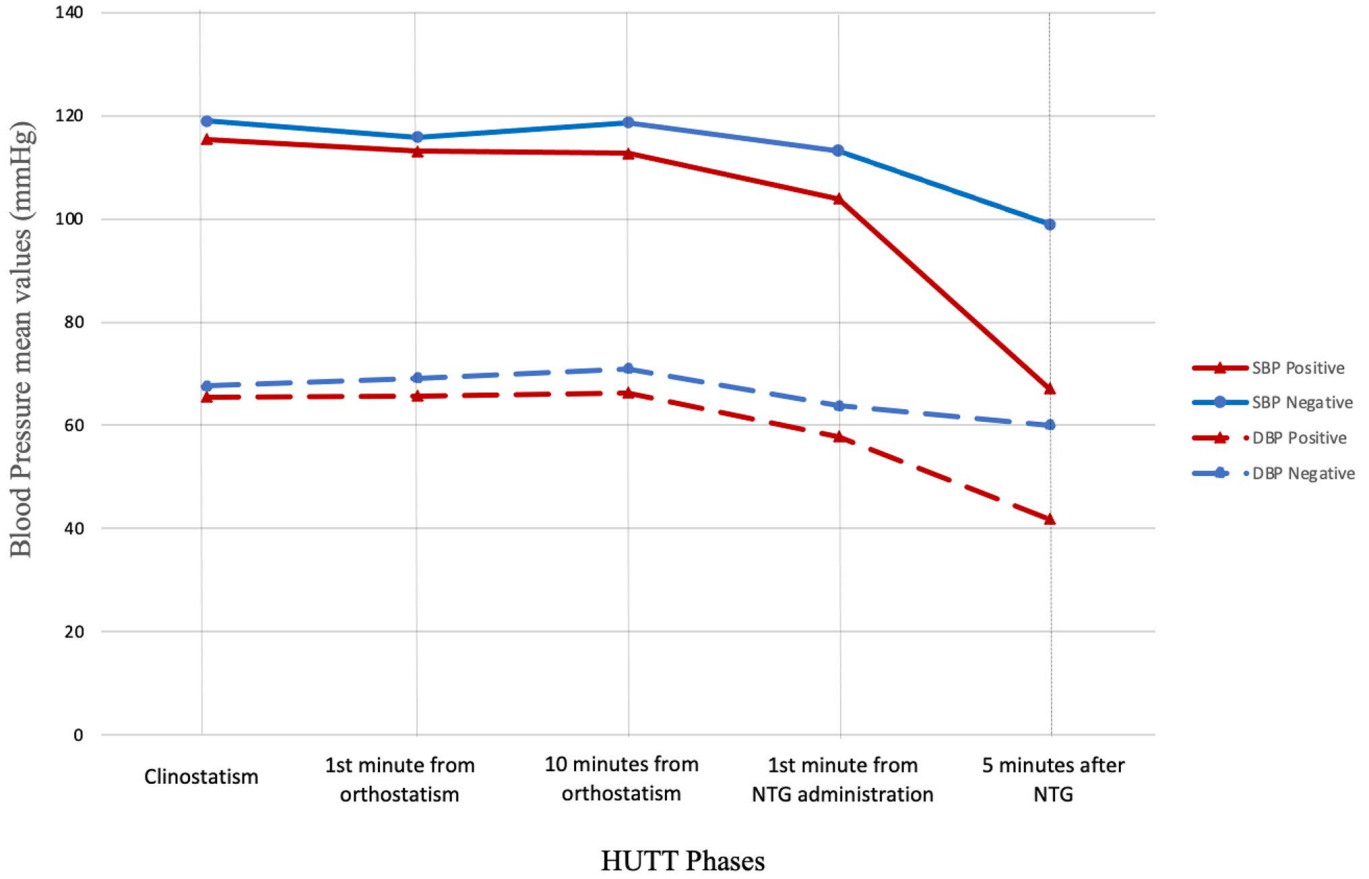
Systolic and diastolic blood pressure trends during HUTT between patients with positive and negative response

**Fig. 2:**
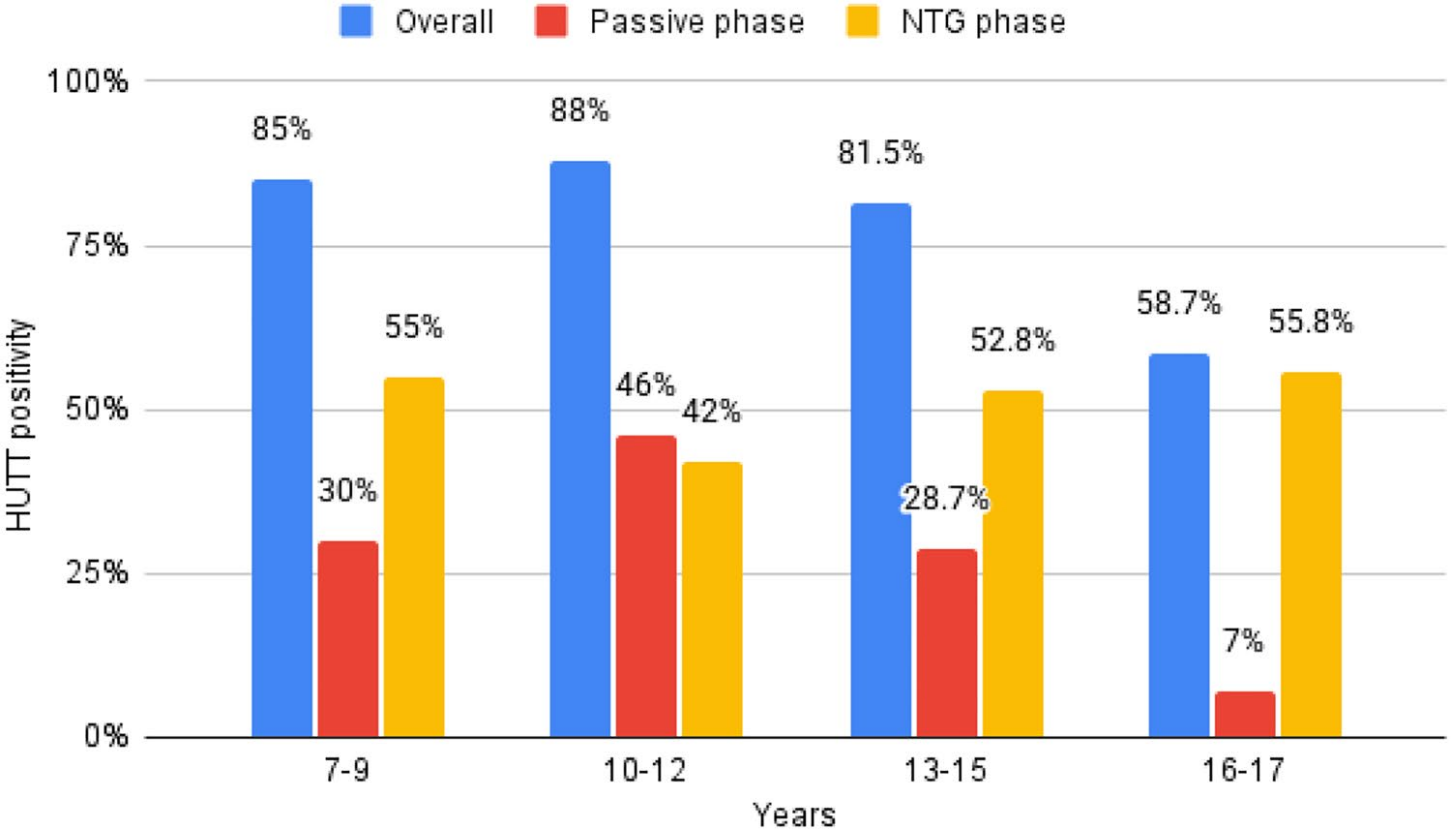
Age distribution of head-up tilt test (HUTT) positivity stratified by HUTT phases

Patients with a positive HUTT response were younger (14 ± 2.8 versus 15.7 ± 2.4 years; *p* < 0.0001) and most likely female (61.7% versus 45.5%; *p* 0.001). At baseline, they exhibited lower systolic blood pressure (SBP) (115.4 ± 9.5 versus 119 ± 13; *p* = 0.01), higher heart rate (HR) (82.2 ± 15.1 versus 77.5 ± 18.9; *p* = 0.03), less frequent history of non-classical (15.2% versus 42.8%; *p* < 0.0001) and situational syncope (3% versus 10.4%; *p* = 0.009) than those with negative HUTT response (Table 1). Both systolic and diastolic blood pressure (DBP) mean values were significantly lower throughout the whole duration of HUTT among patients with positive response compared with those with negative response (Fig. 1). The age distribution of HUTT positivity stratified by the HUTT phases was shown in Fig. 2.

At multivariable analyses, age (OR: 0.84; *p* = 0.005), female sex  (OR: 2.3; *p* = 0.005), history of non-classical syncope (OR: 0.23; *p* < 0.001), and situational syncope (OR: 0.2; *p* = 0.006) remained independently associated with HUTT positivity (Table 3).

**Table 3:** Univariable and multivariable analysis for clinical characteristics associated with HUTT positivity

	Univariate		Multivariate	
	OR (95% CI)	*p*-Value	OR (95% CI)	*p*-Value
Age	0.78 (0.7–0.88)	< 0.001	0.84 (0.74–0.95)	0.005
Female	1.94 (1.15–3.26)	0.013	2.3 (1.29–4.11)	0.005
Smoker	3.1 (0.39–24.8)	0.29	–	–
BMI	0.96 (0.78–1.17)	0.66	—	
SBP (recumbent)	0.97 (0.94–0.99)	0.01	1 (0.97–1.03)	0.83
DBP (recumbent)	0.98 (0.95–1)	0.96	—	—
HR (recumbent)	1.02 (1–1.04)	0.37	1 (0.98–1.02)	0.86
Number of syncope before HUTT	0.99 (0.9–1.09)	0.89	—	—
Traumatic syncope	1.34 (0.59–3.06)	0.48	—	—
Nonclassical	0.24 (0.13–0.43)	<0.001	0.23 (0.12–0.43)	< 0.001
Situational syncope	0.27 (0.09–0.77)	0.015	0.2 (0.07–0.64)	0.006

## Discussion

The main findings of the present study can be summarized as follows: 1) The NTG-potentiated HUTT positivity rate among the pediatric population was 74.9%, ranging from 51.5% to 81.6% among patients with non-classical and classical vasovagal syncope, respectively; 2) the specificity was 81.3%; 3) patients with a positive HUTT response showed lower blood pressure values throughout the entire duration of the HUTT compared with those with negative response; 4) younger age and female sex  were independent risk factors for HUTT positivity; 5) non-classical presentation and situational syncope decreased the probability of HUTT positivity; (6) the cardioinhibitory response was more frequent among patients with non-classical presentation.

### Diagnostic value

The HUTT positivity rate of 81.6% among patients with classical VVS may be considered the real sensitivity of NTG-potentiated HUTT in the pediatric population since a positive HUTT supports a likely diagnosis of VVS when associated with a typical clinical presentation. This value was significantly different from the positivity rate of 18.7% found in our healthy pediatric controls and indicated a good diagnostic accuracy in the pediatric population. These data were fairly similar to those found in the adults, where NTG-potentiated HUTT demonstrated a sensitivity of 81.5% [4]. The specificity was lower in the pediatric population compared with the adult population when using NTG-potentiated HUTT (81.3% versus 92%) [9, 12].

Overall, the diagnostic accuracy of HUTT in the pediatric population was similar to that found in the adults. In literature, the diagnostic value of HUTT among pediatric patients has been questioned by some authors owing to the high rate of false-positive responses [13, 14]. These results might be related to the use of isoproterenol protocols, which were associated with a reduction in HUTT specificity when compared with those with sublingual nitrates [3]. HUTT positivity rate among patients with a non-classical syncope was much lower, about 51.5%. A lower positivity rate of HUTT in patients with non-classical syncope has been previously reported [15], suggesting a non-neurogenic mechanism of loss of consciousness, warranting further investigation. Among the pediatric population, the HUTT positivity rate during the orthostatic passive phase was two-fold higher than those reported in the adults (22.5% versus 11.6%) [4]; this evidence might be explained by the different cardiovascular adaptation patterns, owing to immature venoconstriction responses and lower muscle mass. [16].

### Impact of baseline hemodynamic values on HUTT positivity rate

Patients with a HUTT positive response had lower SBP and higher HR than those with a negative HUTT. This finding is consistent with results observed in the adult population [17, 18] and suggests that, even in pediatric patients, a low BP value is an important trigger of the neurally mediated reflex. The above findings suggest that different hemodynamic features are already evident under basal conditions: our interpretation is that a reduced venous return and stroke volume, reflected by a lower SBP already present at rest, constitute the primary pathophysiological mechanism in patients prone to reflex syncope. The reduced venous return and stroke volume is accompanied by compensatory increases in HR.

### Clinical predictors of HUTT positivity

There are few and contrasting data about the impact of age and sex  on HUTT positivity rate. Previous studies [19, 20], not exclusively focused on pediatric patients, showed a decreasing trend in HUTT positivity with increasing age. This finding may be explained by the immature autonomic system and altered baroreceptor sensitivity in the early stage of life, which contribute to an increased susceptibility to vasovagal syncope [21].

Regarding the  sex-related impact, some previous studies demonstrated higher prevalence of positive responses in female patients [20, 22]. Among our population, female sex  was an independent predictor of HUTT positivity in the overall population, increasing the risk of a positive response by about two times. The sex-related differences in blood pressure values may explain these results. In our study population, female patients showed lower blood pressure values compared with male patients at baseline and throughout the entire HUTT duration, extending previous evidence from the general population to the pediatric population [17].

No data are available about the association between clinical presentation of syncope and HUTT responses in pediatric patients since all studies are limited to the adults [4]. Among our study population, the non-classical syncope was inversely correlated with HUTT positivity. Non-classical syncope characterized by the absence of prodromal symptoms or clear triggers may reflect a different underlying mechanism that is distinct from the typical neuro-mediated reflex pathway. However, the HUTT was able to reproduce the spontaneous symptomatology in about 50% of the cases, leading to a diagnosis of atypical vasovagal syncope. Among them, HUTT was useful to detect the cardioinhibitory form, which had the highest prevalence, as previously demonstrated in adults. Cardioinhibitory reflex syncope can often present without (or with very short) prodromal symptoms [23]. Understanding the pathophysiological mechanisms of syncope is of pivotal importance for optimized personal therapy: younger patients with severe and recurrent episodes of cardioinhibitory vasovagal syncope may benefit from fludrocortisone or midodrine [24] or direct treatments such as cardiacneuroablation since pacing is contraindicated for this population according to the latest guidelines. [11, 25].

Situational syncope was also inversely correlated with HUTT positivity among our population. In our cohort, this condition was poorly represented, with no cases in the preschool-age group and most of them occurring during adolescence. Previous studies on older populations have shown that situational syncope becomes more prevalent with age and is often associated with a high number of comorbidities [26]. Situational syncope in the pediatric population may represent a “once-in-a-lifetime-event,” which could explain why it emerged as a protective factor against a positive HUTT result in our analyses. This suggests that isolated episodes of situational syncope in children may not indicate a broader susceptibility to orthostatic intolerance.

### Limitations

Our results should be interpreted considering the limitations related to the retrospective, observational nature of the study. The classification of patients into classical and non-classical syncope may be affected by challenges in obtaining clinical histories in a pediatric population. The relatively small number of patients in the non-classical syncope group may have limited the statistical power of the analyses. Even if HUTT is not necessary when the diagnosis of reflex syncope is certain/highly likely after the initial evaluation, we suggest performing it when the identification of hemodynamic features is needed to optimize the treatment.

## Conclusions

The NTG-potentiated head-up tilt test showed a high positivity rate, sensibility, and specificity among pediatric patients with suspected reflex syncope. Our findings were consistent with those found in the adult population and support the use of HUTT in the pediatric population as well as in adults. Female sex and younger age are independent clinical predictors of HUTT positivity; in contrast, non-classical presentation and situational syncope were inversely correlated with HUTT positivity. The prevalence of HUTT-induced cardioinhibitory response was high among patients with a non-classical presentation of syncope.
